# Supplementary material for: Content-rich biological network constructed by mining PubMed abstracts
Source: BMC Bioinformatics. 2004 Oct 8;5:147. doi: 10.1186/1471-2105-5-147 (PMC528731; doi:10.1186/1471-2105-5-147)
Supplement: Additional File 2 — The original results of the above study (non-essential files are deleted to keep the file size under the limit set by BMC bioinformatics). [file 1471-2105-5-147-S2.bz2 › chilibotAdditionalFile2/dip05/171ID9388212E586/html/IL6ST_JAK2.html]

 


 **IL6ST** and **JAK2** 
  
Found 57 abstracts in PubMed, retrieved 05.  
 

 What does Google say? 
 PDF only 
| .edu only 

---

**Interactive relationship** (e.g. stimulation, inhibition, etc)

**Inhibitory relationship**- Importantly, tyrosine phosphorylation of  **JAK2**  is reduced in the face of increased gp130  [ **IL6ST** ]  phosphorylation, indicating impaired downstream activation of this critical pathway in DCM.  Ref: 12591746 Circulation, 2003
**Neutral relationship**

**Non-interactive relationship** (e.g. studied together, co-existance, homology, etc.)

- Inactivation to CNTF was not due to a decrease in CNTF receptor subunit gp130  [ **IL6ST** ]  or in levels of Jak1 or  **Jak2** .  Ref: 11882386 Cell Signal, 2002
- RESULTS We showed that  **JAK2** , STATs, 5a and 6 and gp130  [ **IL6ST** ]  proteins are tyrosine phosphorylated as early as 5 30 min post MI.  Ref: 12504822 Cardiovasc Res, 2003
